# Peer Mentoring as a Tool for Developing Soft Skills in Clinical Practice: A 3-Year Study

**DOI:** 10.3390/dj9050057

**Published:** 2021-05-17

**Authors:** Antonio M. Lluch, Clàudia Lluch, María Arregui, Esther Jiménez, Luis Giner-Tarrida

**Affiliations:** 1 Integrated Dentistry Department, Faculty of Dentistry, Universitat Internacional de Catalunya, 08195 Sant Cugat del Vallés, Spain; alluch-alumni@uic.es; 2 Pediatric Dentistry Department, Faculty of Dentistry, Universitat Internacional de Catalunya, 08195 Sant Cugat del Vallés, Spain; claudialluch@uic.es; 3 Dentistry Department, Faculty of Dentistry, Universitat Internacional de Catalunya, 08195 Sant Cugat del Vallés, Spain; lginer@uic.es; 4 Faculty of Education, Universitat Internacional de Catalunya, 08195 Sant Cugat del Vallés, Spain; ejimenez@uic.es

**Keywords:** dental education, mentoring, non-technical skills training

## Abstract

Education currently focuses on improving academic knowledge and clinical skills, but it is also important for students to develop personal and interpersonal skills from the start of their clinical practice. The aim was to evaluate the effect of peer mentoring in third-year students and to gauge the evolution of non-technical skills (NTS) acquisition up to the fifth year. The study groups were selected between September 2015 and May 2018, based on the NTS training they had or had not received: (1) fifth-year students with no training (G1); (2) third-year students mentored in NTS (G2a); and (3) a small group of fifth-year students who became mentors (G2b). A total of 276 students who took part in this study were assessed using a 114-item self-evaluation questionnaire. Data were collected from seven surveys conducted between September 2015 and May 2018, and statistical analysis was performed using one-way ANOVA and Fisher’s post-hoc test. G2a improved their non-technical skill acquisition over three years of clinical training up to their fifth year. This group and G2b showed statistically significant differences compared to non-mentored students (G1). Peer mentoring at the beginning of clinical practice is a valid option for training students in non-technical skills.

## 1. Introduction

In the last decade, one of the most controversial and widely discussed topics in higher education is the development of soft or non-technical skill education [1]. In the case of health science education, the current focus is on improving academic knowledge and clinical skills improvement [2]. Both in dentistry and other health science disciplines, the dentist–patient relationship is not only technical but also based on emotional interactions [3]. This second skill is known as a non-technical skill.

Non-technical skills (NTS) can be defined as a set of social skills, including soft skills, that are perfectly compatible with technical skills, that help health professionals adapt to their environment, and that allow them to perform their work efficiently [4,5]. In medicine, dentistry has also been introduced in recent years to create a model of education that is more holistic and integrated [5]. NTS have been found to improve technical skills in professions like aviation that have been applied for many years, which include situational awareness, decision making, teamwork, leadership, and stress and fatigue management [4]. In terms of effective dental practice, both technical and non-technical skills are essential [2,6].

NTS include soft skills (i.e., social skills), which were first defined in the early 90s. Salovey and Mayer [7] defined emotional intelligence as a form of social intelligence used to control one’s own emotions and those of others to discriminate between them and to harness this information to guide one’s thoughts and actions. Furthermore, soft skills help people adapt to their professional and personal life to overcome new challenges [1]. Over the years, studies on emotional intelligence in health care professionals have grown exponentially [8].

The transition between preclinical and clinical practice is one of the most critical moments in dental studies [9]. The first experience in the clinical environment entails new responsibilities, as well as clinical and legal protocols that students must acquire to manage their work, interact with patients, and work as a team. Verbal and nonverbal communication is an essential element of the patient–dentist relationship [10]. An important part of this involves developing verbal communication [11] and listening skills, which help patients understand their oral health condition and needs [6]. The students’ empathy and assertiveness are essential to this end [12], as are identifying and managing both their emotions and those of others [8]. Additional relevant basic skills for clinical practice include reasoning, problem-solving, decision-making, using and assessing information, and showing self-confidence. Unlike technical skills, non-technical skills in self-management, work management, and interrelationship skills enable students to think critically and are the key learning outcomes that further contribute to success or failure [13]. These skills are closely tied to safe and effective clinical practice centered on the patient and the clinical team. Indeed, providing appropriate and responsive care and listening to patients and respecting their choice, privacy, and dignity [14,15] is no less important than heading a clinical team to perform the best clinical practice [16].

Several studies have associated high levels of emotional intelligence and non-technical skills not only with strong academic performance and more satisfactory clinical results [8,17,18,19] but also with team work management and good rapport between students and their colleagues and staff members [6]. Despite the increasing importance of these skills, as described in the Profile and Competencies for the European Dentists (approved in 2009 by the General Assembly of the Association for Dental Education in Europe), they continue to receive inadequate attention in the curricula [20].

Nowadays, the term mentor is synonymous with a counsellor, guide, or even a coach [21]. Among the different definitions of mentoring in the literature, the most widespread is “a voluntary and reciprocal interpersonal relationship in which a person’s acknowledged expertise shares their experience and learning with a less experienced person, the mentee” [21,22,23]. Depending on the program and the university, a mentor could be a faculty member or a senior student [24,25,26].

The importance and benefits of peer mentoring obtained in clinical training are well documented. These benefits are reaped by mentee and mentor alike [21,25,27,28,29], and they can be an effective pedagogical strategy to promote non-technical skills among students [30]. This experience helps to reduce anxiety, improve confidence, and create a more positive and productive learning experience [31]. However, some authors have identified drawbacks, including opposing perspectives of mentees and mentors and personality clashes that might lead to envy [23]; another example is an unequal distribution in the number of mentors and mentees, or that some mentors might focus on one non-technical skill only, which could leave mentees at a disadvantage compared to peers who develop all these skills [22].

Some authors have highlighted the need to train students as potential university staff [22,24,32]; student mentor training in non-technical skills could be a good step in this direction. Indeed, some studies have underlined the importance of such programs to complement the training of junior faculty staff in addition to leadership programs [23,26,27].

This study analyzed the level of NTS acquired by students between September 2015 and May 2018, based on data collected from third-year students starting clinical practice under the mentorship of fifth-year students trained in NTS. Subsequently, data were obtained from the same students’ NTS training at the end of each academic year from 2016 to 2018.

The aim of this study was to evaluate the outcome of peer mentoring in third-year students and their uptake of NTS across three years, as well as compare the NTS acquisition in fifth-year students, some of whom were mentored in their third year, some of whom received no mentoring, and some of whom became mentors. The research hypothesis was to evaluate whether third-year peer mentoring during clinical practice is a valid tool for students to develop these skills.

## 2. Materials and Methods

The study was approved by the University’s Research Ethics Committee. The head of the mentor program, in collaboration with the university’s Training, Counselling, and Coaching Department, drew up a strategic soft skills framework based on three different models [2,3,6] that are used to promote NTS in the integrated clinical department. Table 1 describes the five groups of NTS studied, according to their different characteristics.

With a view to recruiting fourth-year students who would become mentors in their fifth year, an information session was held in which the objectives of the mentor program were explained. For the selection criteria, the interested students were asked to submit a motivation letter and to pass a personal interview. The exclusion criteria were students with pending subjects or those spending part of the academic year on an exchange program. The students were selected at a ratio of 1 per 4 pairs of third-year students, which was proportionally equal to the clinical faculty.

**Figure 1:**
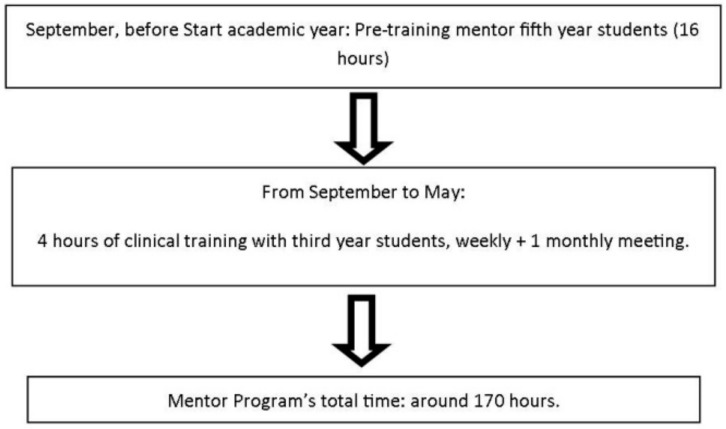
Mentor program framework.

The program was divided into three parts in which the mentors worked either together or individually depending on the training; as such, by the end of the course they had worked for about 170 h (Figure 1). At the beginning of the academic year, the students underwent 16 h of intensive training comprising communication skills with patient simulation activities, work management, relationship skills, and a group-coaching session to strengthen the importance of teamwork. During this intensive training, students also took a psychometric test to assess their self-knowledge development and to provide a starting point for individual coaching sessions to develop their self-management skills. This initial training and newly acquired knowledge taught mentors how to help third-year students develop and improve their non-technical skills for 4 h a week in a clinical environment, helping them to optimize their time and work in clinical situations like learning how to take radiographs or write up a clinical history using templates. The mentors also showed mentees how to communicate with patients in a clear and comprehensive manner. The last part of the program involved monthly group meetings, in which the mentors discussed their experiences and solved problems together with the help and supervision of faculty members who attended the meetings.

**Figure 2:**
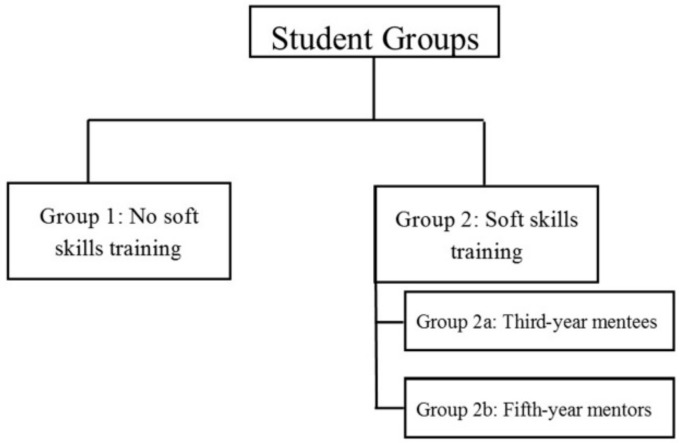
Student distribution.

The students were recruited between September 2015 and May 2018 and divided into two groups according to the NTS training they had received or not. Group 1 (G1) comprised students who did not receive NTS training; Group 2 (G2) consisted of students trained in NTS, who were subdivided into two different groups (Figure 2): G2a were third-year mentees trained in NTS, and G2b were third-year mentees who received additional NTS training in their fifth year in order to mentor third-year students.

**Figure 3:**
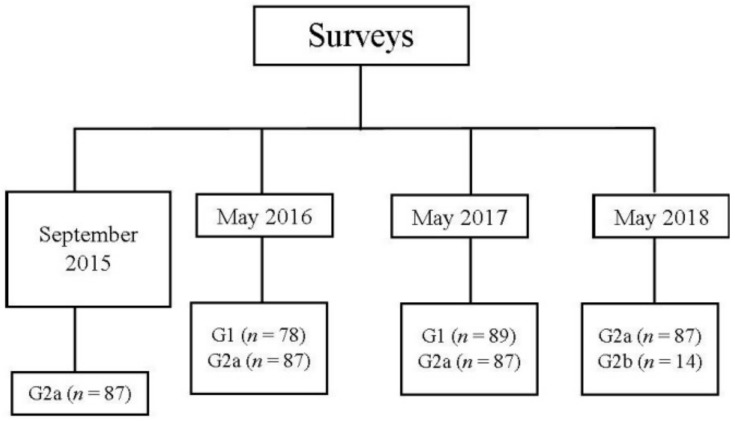
Data survey collection process.

All the students were informed of the purpose of the study, data protection, and possible risks and benefits. The students were then asked to take an online survey at the faculty. More than 90% of the students and 100% of the mentors completed the survey in person. Data collection was carried out electronically between September 2015 and May 2018 (Figure 3). The survey was composed on 114 self-assessment questions divided into the strategic NTS framework described in Table 1. The students scored the questions on a scale of 1 to 10.

Data for G1 was collected at the end of the 2015–2016 and 2016–2017 academic years. G2a students filled in the survey on four different dates: in the 2015–2016 academic year, in September (before clinical practice) and in May, and at the end of the 2016–2017 and 2017–2018 academic years. G2b took the survey only once, upon concluding their studies in May 2018.

All the data was collected in Excel files (Microsoft, Redmond, WA, USA), and statistical analysis was performed using the StatGraphics Centurion XV software (StatPoint Technologies, Inc., Warrenton, VA, USA). The data were analyzed with one-way ANOVA, and differences across groups were identified using the Fisher post-hoc test. The level of statistical significance was set at *p* < 0.05, with a 95% confidence interval.

## 3. Results

Two hundred and seventy-six students took part in the study and were divided in the following groups: G1 (n = 175) subdivided in May 2016 (n = 78) and May 2017 (n = 89), and G2 (n = 101) subdivided in G2a (n = 87) and G2b (n = 14); the last group was the mentors. The survey was filled in different moments, as shown in Figure 2. The students mentored in their first clinical training year showed an evolution in their acquisition of NTS from when they started training under mentor supervision in 2015 until they finished in 2018. One-way ANOVA showed statistically significant differences (*p* < 0.05) in the whole comparisons, but when the data were analyzed with the Fisher post-hoc test, the highest significant differences were between the students starting their clinical training in 2015 and finishing the whole clinical program three years later in May of 2018 (Table 2).

The highest change observed in soft-skills acquisition was in cognitive self-management, and the lowest in the connection relationship. Table 3 shows the scores of the different skills measured by year.

According to one-way ANOVA, group analysis upon completion of clinical training showed statistically significant differences (*p* = 0.02), but the Fisher post-hoc test indicated that G2a and G2b obtained similar scores, showing no statistically significant differences between them (Table 4). It should be noted that these two groups were mentored in their first clinical year. In this context, the statistical differences were between students who never were mentored (G1) and those who were mentored during their first year of clinical training (G2). It should be noted that, even though in the G2 group there was no statistical differences between being a mentor or not in fifth-year, the group of mentors were those who obtained the highest scores.

Analysis of the non-technical skills acquired by the different groups showed that the mentors obtained the highest scores in all the skills when compared with the skills obtained by the other two groups, particularly G1 (non-mentored students). While the three groups obtained the lowest scores in cognitive self-management, the highest score differed depending on the group: the non-mentored students and the mentees scored the highest for connection relationship, while the mentors scored the highest for work management (Table 5).

## 4. Discussion

The peer mentoring program was designed to facilitate the incorporation of third-year students in NTS acquisition during their clinical practice through mentoring by fifth-year students. The idea for implementing this program and introducing NTS training during clinical practice arose from the need to train dental students in these skills and ensure that the curriculum that is more focused on technical skills. The results of the present study showed that both mentors and mentees improved their NTS in clinical management, particularly in terms of relationships and self-management. Mentor’s expertise is developed throughout their clinical training and reinforced by NTS training during the program.

In order to reduce students’ stress and to increase their self-confidence, several universities that run training programs centered on NTS acquisition have stressed the importance of conducting this training alongside clinical practice [10,25,30,33]. However, some of these studies focused on one specific NTS, the most common being communication [10,11,30], followed by teamwork [30]. In the belief that all non-technical skills have equal relevance in clinical training, the authors of the present study analyzed all of them. The results showed an improvement in mentors and mentees when compared with non-mentored students, above all in self-management, relationship, and teamwork, in accordance with other studies [30,34].

To improve their skills, the mentors prepared and developed some clinical aids (videos, pictures, or templates) to show mentees how to organize working time, fill in clinical records, prepare the work area, conduct X-ray management, and fill in the periodontal card. They also improved their communication skills explaining all these procedures to mentees, and other abilities such as solving problems or making decisions when they counselled third-year students in patient management, and showed them strategies to speak with patients. This system is more centered on an in situ clinical scenario than other systems described in the literature that combined portfolio development with clinical recommendation and supervision by a mentor faculty member [33]. In the present study, when comparing the evolution of three years of mentees, the authors showed an improvement in their non-technical skills. It is important to highlight that they only received a mentor’s support in their first year of clinical training, but the acquired knowledge was well assimilated. This outcome could be related to the view of Alzahem et al. [9], who stated that supervision at this critical juncture reduces stress levels and enhances technical and non-technical skills. In addition, it was observed that by the time they reached the final year of clinical practice (i.e., based on results from May 2018), although the mentors received extra training, there was not much difference between them and the rest of their peers who were not trained in this type of skill. However, in contrast, the systematic review by Nicolaides et al. [5] highlights the need for regular training of non-technical skills because it has been found that these skills diminish over time. Therefore, it is important to introduce these types of skills into the curriculum in combination with clinical skills for the benefit of all students. The professional development of students’ clinical skills and knowledge should not be underestimated.

While some universities used faculty staff as mentors [23,26,28,33], the facilitators of the mentor program opted to use a group of fifth-year students, with whom third-year mentees felt more at ease when discussing their doubts. Hence, mentors must undergo specific training before starting the program. The decision to assign 16 h of mentor training conforms to the average training time of between 2 and 35 h, as described in the systematic review conducted by Carey et al. [10]. The training observed in the present study was supplemented by monthly meetings in which the mentors consolidated their training and expressed any problems or doubts concerning their mentees. These meetings also served to encourage teamwork and to avoid the problems of calibration described in some studies [9,33]. Furthermore, these meetings allowed the facilitators to mentor the students in a similar way to how the literature described mentoring and leadership programs to train new staff members [23,26]. While coaching sessions for mentor students in dentistry have not been reported in the literature, the facilitators considered it important to use a professional coach from outside the dentistry profession to reinforce and empower mentors as future clinicians and as people. The results of this study showed that student mentors obtained the highest scores in NTS, especially in emotional self-management and work management, a finding that could relate to the additional training. These results are in accordance with the description of Nicolaides et al. [5], as they stated that NTS could be a catalyst at an individual improvement level, which would allow for better organization of multidisciplinary teamwork.

Since its inception, the program has seen an increase in the number of students applying to become mentors, in accordance with the findings of Stenfors-Hayes et al. [35]. This kind of program shows that mentors are suitably trained for inclusion in the faculty staff [24,32,35] or used to complement junior staff training courses [23,26]. At present, some of the mentors who took part in the program under this study joined the clinical faculty.

The limitations of the present study are related to the study plans and the schedules of mentors. It was challenging to find suitable times for monthly meetings that avoided class times or were compatible with clinical trainings, which is consistent with findings in other studies [30]. In addition, given the facilitators’ need to adapt their own schedules to those of the students, they need to consider the cost benefits of the program (as discussed in Romanelli et al. [19] and Stewart et al. [31]). Future perspectives could involve the study of new faculty staff’s NTS, with or without prior preparation in this field, as well as whether third-year students improve their development of NTS with or without mentor assistance. Patients’ views on the non-technical skills of students’ post-treatment should also be investigated in future research.

As a consequence of the health situation caused by COVID-19, we will have to consider whether part of the NTS training should be done online for the mentor group, as it is important to keep the training of the 3rd year students in the clinical environment. If this is not possible, online strategies should be explored to help students learn these skills outside the clinical scenario by solving clinical cases and role-playing sessions online, as other universities have done during the duration of the pandemic situation [36,37,38].

## 5. Conclusions

Due to the clinical faculty’s difficulties in simultaneously training third-year students in technical and NTS, peer mentoring among students is a valid option for NTS training. The results showed that mentees improved their confidence and development of the acquired NTS without specific mentoring. Furthermore, mentors increased their NTS training in clinical practice, which could be a factor in their incorporation into clinical faculty.

## Figures and Tables

**Table 1:** Strategic non-technical skills framework.

Non-Technical Skills	Characteristics
Cognitive self-management	Data collection
	Analysis
	Synthetic thinking
	Logical judgment
	Reliability
Emotional self-management	Autonomy
	Mental resistance
	Self-confidence
	Perseverance
Connection relationship	Empathy
	Assertiveness
	Active listening
Impact relationship	Conflict management
	Credibility
	Communication
Work management	Problem solving
	Precision
	Planning and organizing
	Orientation in the objectives

**Table 2:** Mean and standard deviation (SD) of mentored students (G2a).

Survey Time	Mean (SD)
September 2015	67.17 (4.23) ^a^
May 2016	76.38 (3.14) ^b^
May 2017	78.57 (2.51) ^bc^
May 2018	81.20 (1.40) ^c^

Superscript letter in the same column showed significant statistical differences (*p* < 0.05).

**Table 3:** Mentee acquired skills.

Non-Technical Skills	September 2015	May 2016	May 2017	May 2018
Cognitive self-management	60.91	72.02	75.36	79.13
Emotional self-management	67.62	76.57	76.83	81.09
Connection relationship	72.14	80.09	81.78	83.07
Impact relationship	69.49	78.4	79.48	81.41
Work management	65.71	74.82	79.38	81.28
Total	67.17	76.38	78.57	81.20

**Table 4:** Mean and standard deviation (SD) in 5th year students.

Group	Mean (SD)
G1	76.99 (3.51) ^a^
G2a	81.20 (1.40) ^b^
G2b	83.86 (1.47) ^b^

Superscript letters denote statistically significant differences (*p* < 0.05).

**Table 5:** Comparison of non-technical skills acquired by students in this study.

Non-Technical Skills	G1 2016–2017	G2a May 2018	G2b May 2018
Cognitive self-management	72.01	79.13	81.89
Emotional self-management	76.86	81.09	84.37
Connection relationship	81.01	83.07	83.78
Impact relationship	79.49	81.41	83.32
Work management	75.56	81.28	85.92
Total	76.99	81.20	83.86

## Data Availability

Not applicable.
